# The Size of Spontaneous Pneumothorax is a Predictor of Unsuccessful Catheter Drainage

**DOI:** 10.1038/s41598-017-00284-8

**Published:** 2017-03-15

**Authors:** Tung-Ming Tsai, Mong-Wei Lin, Yao-Jen Li, Chin-Hao Chang, Hsien-Chi Liao, Chao-Yu Liu, Hsao-Hsun Hsu, Jin-Shing Chen

**Affiliations:** 1 Department of Surgery, National Taiwan University Hospital and National Taiwan University College of Medicine, Taipei, 10002 Taiwan; 2 Graduate Institute of Epidemiology, College of Public Health, National Taiwan University, Taipei, 10055 Taiwan; 3 Genomic Research Center, Academia Sinica, Taipei, 11529 Taiwan; 4 Department of Medical Research, National Taiwan University Hospital, Taipei, 10002 Taiwan; 5 Department of Traumatology, National Taiwan University Hospital, Taipei, 10002 Taiwan; 6 Division of Thoracic Surgery, Department of Surgery, Far Eastern Memorial Hospital, New Taipei City, 22060 Taiwan

## Abstract

Small-bore thoracic catheter drainage is recommended for a first large or symptomatic episode of primary spontaneous pneumothorax (PSP). However, one-third of these patients require a second procedure because of treatment failure. We investigated the factors associated with unsuccessful pigtail catheter drainage in the management of PSP. In this retrospective study, using a prospectively collected database, we enrolled 253 consecutive patients with PSP who underwent pigtail catheter drainage as initial treatment, from December 2006 to June 2011. The chest radiograph was reviewed in each case and pneumothorax size was estimated according to Light’s index. Other demographic factors and laboratory data were collected via chart review. Pigtail catheter drainage was successful in 71.9% (182/253) of cases. Treatment failure rates were 42.9%, 25.9%, and 15.5% in patients with pneumothorax sizes of >62.6%, 38–62.6%, and <38%, respectively (tertiles). An alternative cut-off point of 92.5% lung collapse was defined using a classification and regression tree method. According to the multivariate analysis, a large-size pneumothorax (*p* = 0.009) was the only significant predictor of initial pigtail catheter drainage treatment failure in patients with PSP. Early surgical treatment could be considered for those patients with a large-sized pneumothorax.

## Introduction

Primary spontaneous pneumothorax (PSP) is defined as the trapping of air in the pleural cavity of patients without underlying lung disease or thoracic trauma. PSP usually affects young and tall male patients, often adolescents with a slim build. The global incidence is 7.4–18/1,000,000 men per year and 1.2–6/1,000,000 women per year^1, 2^. The optimal treatment for patients presenting with a first episode of PSP is controversial and existing guidelines vary^3–5^. The British Thoracic Society (BTS) 2010 pleural disease guidelines recommended needle aspiration (14–16 G) or small-bore (<14 French, 4.7-mm diameter) thoracic catheter drainage as the first-line treatment for patients with a large PSP^3^. However, the results of simple aspiration are not satisfactory for a large portion of patients. First, the success rate of needle aspiration varies widely in the literature, ranging from 38%–85%^6–15^; second, almost one-third of patients need an additional invasive procedure^6^. Therefore, several modified strategies, including thoracostomy with chest tube or pigtail catheter, chemical pleurodesis with sclerosing agents, or video-assisted thoracic surgery are generally required^16, 17^.

The issue of initial treatment failure in PSP is important. An appropriately selected treatment strategy leads to a better outcome and less waste of medical resources. In some patients with a high risk of treatment failure, physicians may choose a more efficient management approach than simple aspiration. Since 1985, several studies focused on this issue, and reported several factors associated with treatment failure with simple aspiration. These factors included age >50 y, pre-existing lung disease, a large volume of aspiration, and pneumothorax size^6, 10, 12, 18, 19^. Harvey *et al.* reported a significant difference in the amount of air aspirated between successfully treated patients and those who showed treatment failure^6^. In a retrospective study with 91 cases, Chan *et al.* reported that treatment was more likely to fail in patients with a large pneumothorax (>40%)^10^. However, these studies were limited by their retrospective nature, small study populations (<100 cases), and heterogeneous patient populations. Furthermore, there have been no previous studies discussing the factors affecting initial treatment failure after catheter drainage.

The aim of our study was to investigate the risk factors associated with treatment failure of PSP treated with a small-bore (8-French) pigtail catheter for thoracic drainage as the initial treatment based on a prospectively collected, single-center database in a large homogenous patient population.

## Results

### Characteristics of patients

From 2006 to 2011, a total of 253 patients were enrolled into the study and underwent trans-thoracic pigtail catheter insertion and drainage. Overall, 182 patients were successfully treated by this method (71.9%; success group) and 71 patients were considered treatment failures (28.1%; failure group). The failure group included 60 cases with persistent air-leaks at 72 hours after the procedure and 11 cases who showed an enlarging pneumothorax on serial chest radiographs after the pigtail catheter was removed. In these 11 cases, the catheters were all removed once there were no ongoing air-leaks.

Table 1 demonstrates the clinical features of the two groups. We carefully recorded every patient’s clinical data with a thorough chart review. Both groups were composed of younger adults (mean age: 22.5 ± 5.5 y) with a slim body shape (body mass index [BMI] = 19.3 ± 2.3 kg/m^2^). A smoking history did not affect the outcome of treatment (*p* = 0.641). In our study, more patients had a pneumothorax affecting the left side (147/253, 58.10%), but there was no significant difference in treatment outcome based on the side of the pneumothorax (*p* = 0.943). There were no significant differences in hemoglobin, white blood cell (WBC) count, and renal or hepatic function between the successfully treated patients and those with treatment failure.

**Table 1:** Characteristics of successfully treated and treatment failure groups.

	Failure (N = 71)	Success (N = 182)	*p* value
Age (years)^*^	22.5 ± 5.5	21.8 ± 5.6	0.380
Sex (male)	64 (90.1%)	161 (88.5%)	0.825^※^
Height (cm)^*^	173.5 ± 7.1	172.4 ± 7.0	0.277
Weight (kg)^*^	58.5 ± 8.8	57.9 ± 8.9	0.643
BMI^*†^ (kg/m^2^)	19.4 ± 2.6	19.3 ± 2.3	0.756
Smoking	24 (33.8%)	56 (30.8%)	0.641
Side involved (left)	41 (57.7%)	106 (58.2%)	0.943
Pneumothorax size^*,‡^	0.60 ± 0.23	0.51 ± 0.21	0.004
Hemoglobin^*^ (mg/dL)	15.0 ± 1.2	15.0 ± 1.4	0.994
WBC (x 10^3^)^*^	8.7 ± 3.0	8.2 ± 2.3	0.204
BUN^*^ (mg/dL)	12.3 ± 2.4	12.7 ± 3.4	0.520
Creatinine^*^ (mg/dL)	1.01 ± 0.13	1.00 ± 0.13	0.572
SGOT^*^ (IU/dL)	21.8 ± 8.5	20.7 ± 7.3	0.417

^*^Mean ± standard deviation. ^†^BMI = body mass index, WBC = white blood cell count, BUN = blood urea nitrogen, SGOT = serum glutamic-oxaloacetic transaminase. ^‡^Estimated by Light index. ^※^Using Fisher exact test.

### Size of pneumothorax and treatment outcomes

In our study, the only factor that had a significant impact on the treatment outcome was the size of the pneumothorax. The treatment failure group had a significantly larger-sized lung collapse than the successfully treated group (60 ± 0.23% vs. 51 ± 0.21%, *p* = 0.004). Table 2 shows the relationship between pneumothorax size and treatment outcome. We split the 253 patients into tertiles according to their initial pneumothorax size, as determined by chest radiography. Based on these measurements, the size of the pneumothorax was classified into three categories based on the severity of lung collapse: massive (>62.6%), moderate (38–62.6%), and mild (<38%) collapse. In patients with a mild collapse, the treatment success rate was 84.52%; while in patients with a massive collapse, the success rate was 57.15%. Further analysis revealed a trend showing that, the larger the size of the lung collapse, the more likely it was that treatment would fail.

**Table 2:** Relationship between severity of pneumothorax size and treatment outcome.

Lung collapse (%)*	Failure	Success
Mild (<38%)	13 (15.48%)	71 (84.52%)
Moderate (38–62.6%)	22 (25.88%)	63 (74.12%)
Massive (>62.6%)	36 (42.85%)	48 (57.15%)

^*^Patients were divided into tertiles according to their initial pneumothorax size, as determined by chest radiography.

We also created an alternative statistical model using the classification and regression tree (CART) method. In this model, all patients were divided into two groups according to the cut-off point of 92.5% lung collapse. Table 3 shows the multivariate logistic regression analysis of the risk factors. Massive collapse (>92.5%) was associated with a higher risk of treatment failure than in other cases, with an OR of 4.23 (95% confidence interval = 1.43–12.47, *p* = 0.0091). Other factors, including age, BMI, and smoking status were not associated risk factors. There were no major complications associated with the pigtail catheter insertion, such as hemothorax, lung penetrating injury caused by the pigtail catheter, or other solid organ injury.

**Table 3:** Multivariate logistic regression analysis of risk factors.

Associated variables	Failure vs. success	
	OR (95% CI)	*p* value
Age	1.03 (0.96, 1.09)	0.44
BMI	0.93 (0.81, 1.07)	0.34
Smoking		
Yes	0.76 (0.36, 1.61)	0.47
No	1	
Pneumothorax size*		
<0.925	1	0.0091
≥0.925	4.23 (1.43, 12.47)	

^*^The alternative cut-off value of pneumothorax size was defined using the classification and regression tree (CART) method.

### Long-term follow-up outcomes

**Figure 1:**
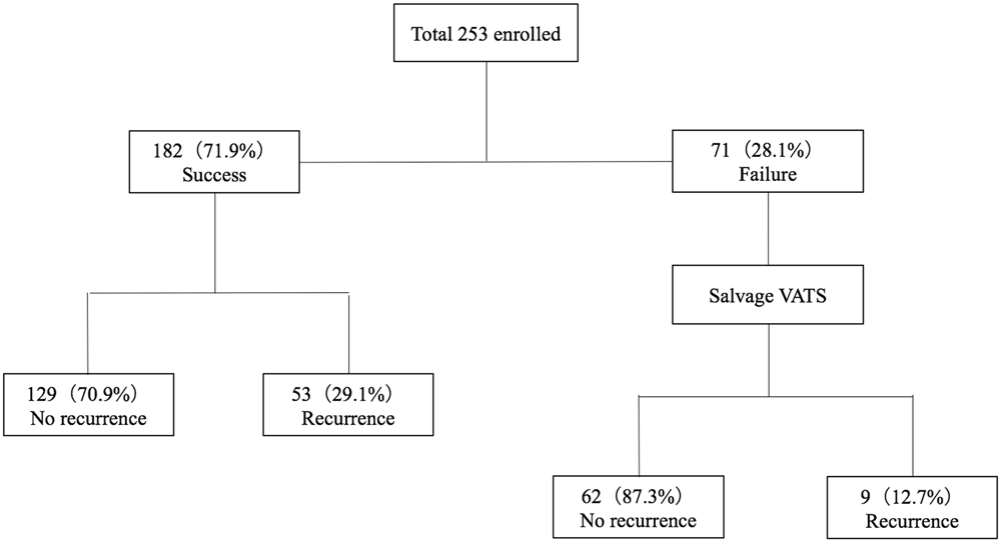
Long-term follow-up results of the study group (n = 253). The mean follow-up time was 90.9 ± 14.6 (60–114) months. *All patients with unsuccessful catheter drainage (n = 71) underwent salvage VATS bullectomy and mechanical pleurodesis.

The long-term follow-up outcomes of the 253 patients were recorded after chart review and phone interview, with a mean follow-up period of 90.9 ± 14.6 (range: 60–114) months. Figure 1 summarizes the results of the long-term follow-up. The recurrence rate in the success group was 29.1% (53/182 cases). The 71 patients with initial treatment failure underwent salvage video-assisted thoracoscopic surgery (VATS, described in the Methods section). The recurrence rate of the patients who received salvage VATS was 12.7% (9/71 cases). Among the patients who underwent salvage VATS, pathological examination revealed that 85.9% (61/71) had bullae, and 12.7% (9/71) had blebs. One case showed emphysematous changes without formation of bullae or blebs.

## Discussion

Pneumothorax remains a challenging disease with a lack of optimal treatment guidelines, especially for those patients experiencing a first episode or “large”-sized pneumothorax. The currently available guidelines, including those set out by the British Thoracic Society (BTS), the American College of Chest Physicians (ACCP), and the Belgian Society of Pulmonology (BSP), all differ in their recommendations for treatment of a large-sized PSP^3–5^. The initial treatment strategies include conservative treatment, simple aspiration, and intercostal pleural drainage with a small-bore pigtail catheter or traditional large-bore catheter^3–5, 16, 17^. Traditional conservative treatment includes bed rest, supplemental high flow oxygen, and analgesics. Both bed rest and high-flow oxygen supplementation improve trans-thoracic air reabsorption^20, 21^. When conservative treatment fails, or is not suitable, invasive treatment, such as the placement of trans-thoracic drains should be considered. Both the BTS and ACCP guidelines recommend simple aspiration or small-bore trans-thoracic drainage as the initial treatment for PSP^3, 4^.

The role of trans-thoracic drainage in PSP is to remove excess intrapleural air and promote apposition of the visceral and parietal pleura, initiating the process of healing of the lung defect. A retrospective study by Chan *et al.* used a homogenous population of 91 PSP patients treated with simple aspiration, and revealed that treatment failure was associated with a pneumothorax with a size ≥40%^10^. However, that study had several limitations, including its retrospective design, the lack of a universal protocol for estimating pneumothorax size, and a low rate of successful treatment (50.5%).

Our study is the largest study of patients undergoing initial PSP treatment to date, and is based on a prospectively collected, single-center database with a homogenous patient population. We employed the Light index, which is an easily implemented method, for the estimation of pneumothorax size. Our study observed that a large-sized pneumothorax is the only factor associated with treatment failure. Patients with a larger pneumothorax are more likely to experience treatment failure.

We recommend trans-thoracic drainage with a small-sized trans-thoracic pigtail catheter instead of simple aspiration in PSP patients with a small pneumothorax. We routinely connect the pigtail catheter to a water-sealed bottle, which acts as a single-bottle underwater seal chest drainage system, thereby providing continuous trans-thoracic air drainage. The effect of drainage can be augmented with low-pressure negative suction (usually −10 to −20 cmH_2_O)^22^.

Patient safety is always a concern, and pigtail catheter insertion is easy to perform and less invasive than other procedures^23^. Additionally, unlike simple aspiration, insertion of the pigtail catheter does not require repeat procedures. The complications of pigtail catheter insertion are few, especially in young and medically uncomplicated populations^24, 25^. With continuous monitoring of the trans-thoracic space, we can monitor when air-leaking resolves and can respond immediately to any emergency conditions, such as a delayed hemothorax.

We acknowledge that there are limitations to this study. First, it is difficult to measure the pneumothorax volume accurately from chest radiographs, which are two-dimensional images, because the pleural cavity is a three-dimensional structure. However, chest radiography is the most common and accessible method to diagnose PSP in most institutes. Therefore, we decided to use chest radiography, rather than chest computed tomography, to estimate the size of the pneumothorax. On the other hand, this means that our findings might be helpful in settings where computed tomography is not readily available. Second, because our institute lacked the appropriate equipment, we did not record the volume of the air-leaks in this study. A large volume of aspirated air may be related to the size of the pneumothorax, and the BTS guidelines use 2.5 L as the limit for simple aspiration because of the likely presence of persistent air leak. Finally, our study is a retrospective-based analysis similar to other existing literature. Further prospective randomized clinical trials with larger study populations are necessary.

We reported our experience with a large cohort of 253 PSP patients who underwent pigtail catheter drainage, and concluded that the size of the pneumothorax is the only prognostic factor associated with treatment outcome in PSP. Trans-thoracic pigtail catheter drainage has several advantages over simple aspiration. In addition, with the advances in thoracoscopic surgery over the past decade, VATS has become a safe intervention for PSP as the first-line surgical intervention or salvage strategy^26–28^. Early thoracoscopic procedures can be considered as a first-line treatment for patients with a large pneumothorax.

## Methods

### Study design and patients

This study was based on the retrospective analysis of a prospectively collected database. From December 2006 through June 2011, a total of 253 patients with a diagnosis of primary pneumothorax matching the eligibility criteria (described in the next section) were recorded in the database of the National Taiwan University Hospital, a university teaching hospital in Taipei, Taiwan. The enrolled patients were first treated with trans-thoracic drainage by pigtail catheter. The trans-thoracic pigtail catheter (8-French, 2.67-mm diameter) was inserted into the side affected by the pneumothorax and patients were categorized into treatment failure and treatment success groups. Treatment failure was defined by the two following conditions: (1) persistent air leaks 72 hours after the procedure or (2) an enlarging pneumothorax on serial chest radiographs after pigtail catheter removal under the condition of no ongoing airleaks. Treatment success was defined as no detectable pneumothorax after the pigtail catheter was removed and a disease-free status maintained for more than one month after catheter removal.

Patient characteristics including age, gender, smoking status (current smokers and ever-smokers were classified as “smokers”, and never-smokers were classified as “nonsmokers”), and side of the pneumothorax at initial assessment were recorded. Clinical parameters, including hemoglobin, WBC count, and renal and hepatic function, were collected from the patient charts.

The Research Ethics Committee of the National Taiwan University Hospital approved this study. Written informed consent was obtained from all subjects before the procedure, and all procedures and methods were performed in accordance with relevant guidelines and regulations.

### Eligibility criteria

Patients were considered eligible for inclusion in the study if: (1) they had their first episode of spontaneous pneumothorax, and (2) the first chest radiograph after the episode revealed that the rim of air in the thorax was >2 cm, compatible with the definition of a large-sized PSP according to the BTS 2010 criteria^3^. Additional inclusion criteria included age between 15 and 40 years, with adequate hematological function (hemoglobin >10 g/dL, absolute neutrophil count >1.5 × 10^9^/L, and platelets >100 × 10^9^/L), and normal renal and hepatic functions (serum creatinine <1 × ULN, serum glutamic-pyruvic transaminase and serum glutamic-oxaloacetic transaminase <2.5 × the upper limits of normal). Patients were excluded if they (1) had underlying pulmonary disease, such as a congenital anomaly or lung fibrosis, (2) had an emergency condition requiring chest tube thoracostomy or surgical exploration, such as hemothorax or tension pneumothorax, (3) had a history of previous pneumothorax, (4) had a history of an ipsilateral thoracic operation, (5) were pregnant or lactating, or (6) had a serious concomitant illness or medical condition.

### Estimation of pneumothorax size

**Figure 2:**
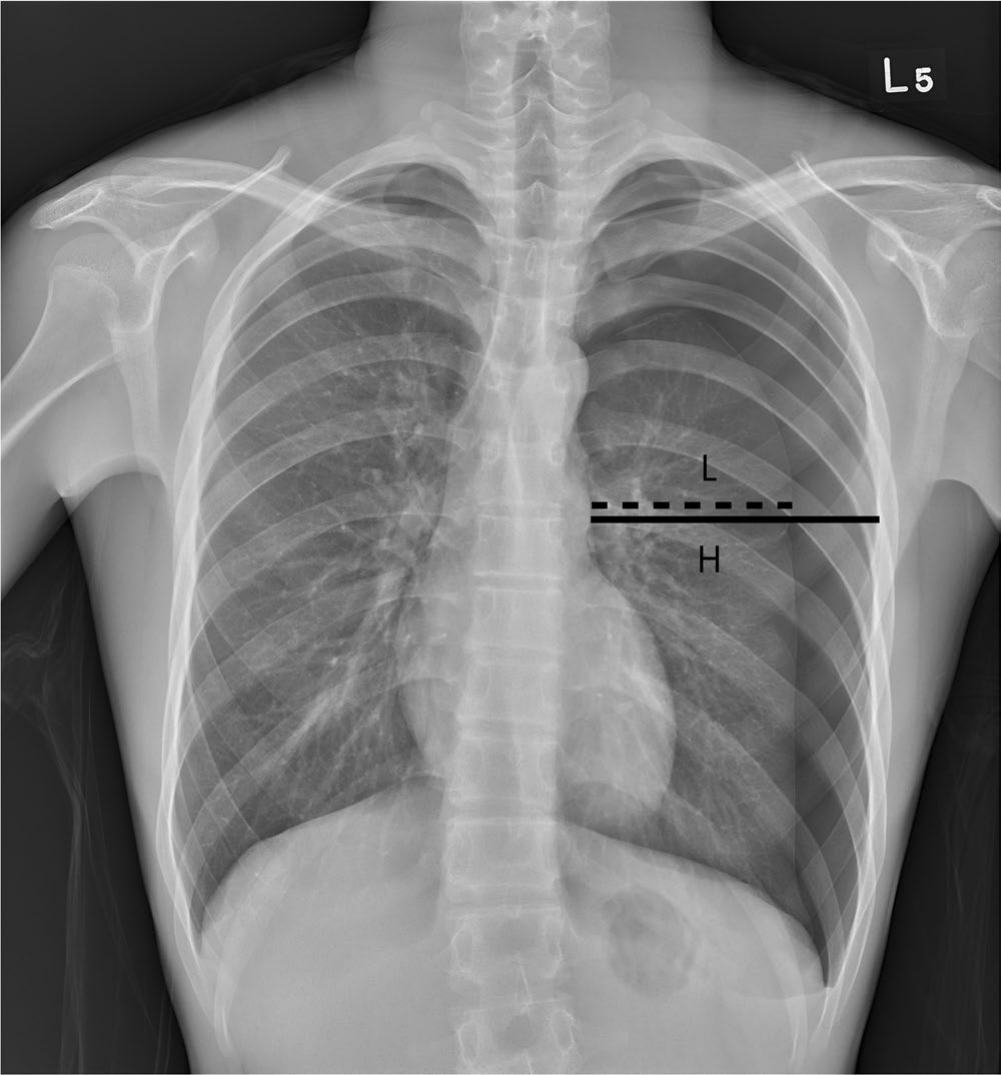
Chest radiograph of a 17-year-old boy with a primary pneumothorax. H represents the diameter of the inner hemithorax at the hilar level, and L represents the diameter of the collapsed lung. Using the Light equation: the size of pneumothorax = (1 − L^3^/H^3^) × 100%, we can easily calculate an estimated pneumothorax size of 34%.

We carefully reviewed the first chest radiograph that confirmed the diagnosis of pneumothorax in every patient. Chest radiographs were retrospectively reviewed by two of the principal investigators (T-M Tsai, M-W Lin, H-C Liao, and C-Y Liu). The investigators were blinded to the patient outcomes, and their decision was recorded after they reached a consensus. We used the Light index^25^, a measurement modality generally accepted in Europe, for the estimation of pneumothorax size. The Light index is based on the proportional relationship between the diameters of a collapsed lung and its hemithorax. The estimated pneumothorax percentage = (1 − L^3^/H^3^) × 100; where H = hemithorax diameter and L = diameter of the collapsed lung (Fig. 2).

### Insertion of the thoracic pigtail catheter

Once the patient was enrolled, thoracic drainage was immediately performed as follows. All procedures were performed by board-certified thoracic surgeons in our institute. Patients were first seated in a semi-supine position and, after skin disinfection and field preparation, a small-bore pigtail catheter (8-French; Bioteque Corporation, I-Lan, Taiwan) was introduced into the first or second intercostal space, at the midclavicular line, after providing local anesthesia with 2% lidocaine. After the catheter had entered the pleural space, it was fixed to the skin using sterile adhesive tape and connected via a three-way valve to a 50-ml syringe. Air was manually aspirated until a resistance was felt and aspiration ceased. The pigtail catheter was then connected to a single-bottle underwater-seal chest-drainage system: a rigid straw is inserted into a sterile chest bottle containing saline solution, with its tip 2-cm below the level of the solution, and the other end of this rigid straw is connected to the pigtail catheter placed in the pleural cavity. Another rigid straw is inserted into the bottle and connected to a negative pressure suction (−10 cmH_2_O) device to control the drainage of the pleural cavity. Chest radiographs were obtained once before the procedure and daily after the procedure in all patients to assess treatment results.

### Operative techniques of salvage VATS

VATS is a better modality than chest tube drainage for the management of unsuccessful aspiration of a PSP^26^. At our institution, the general practical guidelines indicate salvage VATS for patients with recurrence or initial treatment failure of PSP. The procedure is performed in the standard fashion under general anesthesia with double-lumen endotracheal tube intubation, with the patient in a lateral decubitus position with the ipsilateral lung deflated. We use three thoracoscopic ports: one 12-mm and two 3-mm in diameter. A 10-mm 30-degree telescope (Karl Storz, Tuttlingen, Germany) is inserted through the 12-mm port at the 6^th^ or 7^th^ intercostal space, along the middle axillary line. The two 3-mm ports are located in the 3^rd^ or 4^th^ intercostal space, at the anterior and posterior axillary line, respectively. The two 3-mm ports are used as working ports, allowing us to release the pleural adhesions using electrocautery and identify the blebs. Once the blebs are identified, they are held with a 3-mm grasp, and excised with a 45-mm endoscopic stapler through the inspection of another independent 3-mm 30-degree mini-telescope. If no blebs can be identified, blind apical stapling is done at the most suspicious area. To complete the mechanical pleurodesis, the entire apical parietal surface is abraded by inserting a dissector with a diathermy scratch pad through the 12-mm port. Finally, a chest tube (28-French) is placed in the apex area through the 12-mm port site. The surgical specimens are sent for routine pathological examination.

### Statistical analysis

The results were expressed as mean ± standard deviation for continuous variables and frequency (%) for categorical variables. Differences between the failure group and the success group were compared with a two-sample *t*-test for continuous variables, and with the chi-square test or Fisher’s exact test for dichotomous variables. Initially, we divided the patients into tertiles according to their initial pneumothorax size, and performed a univariate analysis of the potential risk factors. To further test the robustness of our data, we created an alternative statistical model using the classification and regression tree method. Using this model, we divided all the patients into two groups by a single cut-off point of lung collapse percentage (92.5%) and performed a multivariate logistic regression analysis. The potential risk factors (*p* < 0.05) in the univariate analysis and some potential clinical confounding factors were included in the multivariate logistic regression model. All statistical analyses were performed using the SAS 9.4 and R 3.3.1 software. Statistical significance was set at *p* < 0.05.
